# Discovery of critical thresholds in mixed exposures and estimation of policy intervention effects

**DOI:** 10.1515/jci-2024-0056

**Published:** 2026-01-09

**Authors:** David B. McCoy, Alan Hubbard, Mark van der Laan, Alejandro Schuler

**Affiliations:** Division of Biostatistics, University of California, Berkeley, USA.; Division of Biostatistics, University of California, Berkeley, USA.; Division of Biostatistics, University of California, Berkeley, USA.; Division of Biostatistics, University of California, Berkeley, USA.

**Keywords:** targeted maximum likelihood estimation, mixtures, interactions, decision trees, 62G08, 62P10, 62H12

## Abstract

Regulations of chemical exposures often focus on individual substances, neglecting the amplified toxicity that can arise from multiple concurrent exposures. We propose a novel methodology to identify critical thresholds in multivariate exposure spaces and estimate the effects of policy interventions that limit exposures within these thresholds. Our approach employs a recursive partitioning algorithm integrated with targeted maximum likelihood estimation (TMLE) to discover regions in the exposure space where the expected outcome is minimized or maximized. To address potential overfitting bias from using the same data for threshold discovery and effect estimation, we utilize cross-validated TMLE (CV-TMLE), which ensures asymptotic unbiasedness and efficiency. Simulation studies demonstrate convergence to the optimal exposure region and accurate estimation of intervention effects. We apply our method to synthetic mixture data, successfully identifying true interactions, and to NHANES data, discovering harmful metal exposures affecting telomere length. Our approach provides a flexible and interpretable framework for policy-makers to assess the impact of exposure regulations, and we offer an open-source implementation in the CVtreeMLE R package.

## 1 Introduction

Assessing the health impacts of multiple, simultaneously occurring chemical exposures – commonly called *mixed exposures* – is a major challenge in environmental epidemiology and public health [1–5]. Existing regulatory frameworks often consider one chemical at a time, overlooking the complex ways that combined exposures can amplify or counteract toxicity [6, 7]. This motivates a search for *threshold-based* interventions that limit exposures collectively, rather than individually, to reduce adverse outcomes and provide interpretable safe limits for multiple exposures.

Numerous approaches seek to evaluate multivariate exposures, including weighted quantile sum (WQS) regression [8], Bayesian mixture modeling [9], and Bayesian kernel machine regression (BKMR) [10]. While each offers insight – for example, capturing nonlinearity or estimating kernel-based dose-response surfaces – they usually either rely on strong model assumptions (directional homogeneity, strict linearity, or specified priors) or do not target a clear *policy-relevant* parameter. In particular, most do not explicitly define or estimate the *causal effect* of restricting exposures to certain thresholds in a high-dimensional mixture space [11, 12].

We propose a novel *data-adaptive* framework for discovering *critical threshold regions* in mixed exposures, together with a *policy-oriented* parameter that quantifies how forcing all individuals into those thresholds would change the population-average outcome. We define the *Attributable Regional Effect* (ARE) as the difference between the observed outcome and the counterfactual outcome under a subregion 𝒜 in the exposure space. Our approach integrates:

1. **Recursive partitioning** of the exposure space via a significance-filtered decision tree. Rather than splitting on predictive variance (as in CART), we split on potential *causal* effects, discarding nodes with sparse data to maintain *positivity*.
2. **Cross-validated targeted maximum likelihood estimation (CV-TMLE)** [13, 14] to generate robust, unbiased estimates of the ARE and valid confidence intervals, even in complex or high-dimensional exposures.
3. **Interpretability for regulation**, since the discovered subregion can be written as simple “cut points,” e.g. $A_{j}\leq \tau_{j}$, which are tangible for policy makers.

We demonstrate in simulations that our data-adaptive procedure identifies true critical thresholds and yields accurate causal estimates of restricting exposures to those thresholds under realistic conditions. We further apply the method to two empirical examples: (i) synthetic mixture data from NIEHS, and (ii) NHANES data on metal exposures and telomere length, revealing interpretable subregions associated with lower or higher health risks. Our CVtreeMLE R package, which has undergone peer review at the *Journal of Open Source Software* [15], implements the methodology for broader use.

The rest of this manuscript proceeds as follows. In Section 2.2, we define the causal parameter (ARE), explain its identification assumptions, and briefly outline TMLE (Section 4). We then present our threshold-discovery algorithm (Section 4), along with theoretical properties in Section 7. Section 8 document simulation results, while Section 9 applies the method to real data. Finally, Section 10 concludes with a discussion of limitations, policy interpretation, and future directions.

## 2 The estimation problem

### 2.1 Setup and notation

We consider an observational study with baseline covariates $\left(W\in R^{p}\right)$, multiple exposures $\left(A\in R^{m}\right)$, and a single timepoint outcome (Y). Let O=(W,A,Y) denote the observable data. We assume that there exists a potential outcome function Y(a) (that is, Y(a) is a random variable for each value of **a**) that generates the outcome that would have been obtained for each observation had exposure been set to A=a. These potential outcomes are not observed, but the observed outcome Y corresponds to the potential outcome for the observed value A of the exposure, that is, Y=Y(A). We use $P_{0}$ to denote the true data-generating distribution. We assume that our $O_{1},O_{2},\ldots ,O_{n}$ are independent and identically distributed (i.i.d.) draws of $O=(W,A,Y)\sim P_{0}$. We decompose the joint density as $p_{Y,A,W}(y,a,w)=p_{Y|A,W}(y|a,w)p_{A|W}(a|w)p_{W}(w)$ and make no assumptions about the forms of these densities. Let μ(a,w)=E[Y∣A=a,W=w] represent the observable (stratum-specific) dose-response function that can actually be estimated from observable data.

Let Ψ map from the set of possible data-generating distributions to the real numbers. Let $\Psi \left(P_{0}\right)$, the value of this map at the true, unknown distribution $P_{0}$, denote a target parameter (estimand) of interest. We can view our observed data ($O_{1},\ldots ,O_{n}$) as a (random) probability distribution $P_{n}$ that assigns a probability mass 1/n to each observation $O_{i}$. Let $\hat{\Psi }$ mapping from empirical distributions to numbers denote our estimator (algorithm) such that $\hat{\Psi }\left(P_{n}\right)$ is our estimate from data.

### 2.2 Defining the attributable regional effect (ARE)

We are interested in estimating the effect of a policy intervention that limits exposures to a specific region $𝒜\subset R^{m}$ of the exposure space. For example, 𝒜 could represent allowable exposure levels that are considered safe. We define a binary indicator variable $T=1_{𝒜}(A)$, where $1_{𝒜}(A)=1$ if A∈𝒜 and 0 otherwise. Let $\pi_{𝒜}(w)=P(A\in 𝒜|W=w)$ be the probability that an observation with covariates w naturally self-selects treatment in the region 𝒜.

We define the modified treatment variable $\tilde{A}$ to represent the exposure distribution once all exposures are forced to self-select within the region 𝒜. The modified treatment has the following density:

$$p_{\tilde{A}|W}\left(a|w\right)=\frac{1_{𝒜}\left(a\right)}{\pi_{𝒜}\left(w\right)}p_{A|W}\left(a|w\right),$$

which preserves the relative self-selection preferences for each available exposure and sets the preferences for “outlawed” exposures to zero. More formally, relative self-selection means that within the allowed exposure region 𝒜, individuals maintain their original exposure preferences relative to each other (conditional on covariates), and exposures outside 𝒜 are set to zero probability. Now we can define the expected population outcome if we were to impose this policy:

$$E[\mu (\tilde{A},W)]=\int \mu (a,w)\text{d}P_{\tilde{A},w}(a,w)\;=\int \mu (a,w)\frac{1_{𝒜}(a)}{\pi_{𝒜}(w)}p_{A|W}(a|w)p_{W}(w)\text{d}a\;\text{d}w\;=\int_{w}\left[\int_{a\in 𝒜}\mu (a,w)\frac{p_{A|W}(a|w)}{\pi_{𝒜}(w)}\text{d}a\right]p_{W}(w)\text{d}w\;=E\left[E\left[Y|T=1,W\right]\right].$$

We have shown that this parameter is a population average of the function Q(T=1,W)=E[Y∣T=1,W] when T is forced to 1. For any value of w,Q(1,w) is a weighted average of the conditional mean outcome across the different exposure levels within 𝒜, thus collapsing it to a single number for each w. We define the *attributable regional effect* (ARE) as

$$\psi_{𝒜}=E\left[Q\left(1,W\right)\right]-E\left[Y\right],$$

representing the difference in the average outcomes if we forced exposure to self-selection within 𝒜 versus the observed average outcome.

### 2.3 Policy interventions and the assumption of relative self-selection

If we do not assume relative self-selection, we would need to account for how each individual’s exposure preferences might change under the policy restriction. For any regulation or restriction, we would need to model the conditional density of exposures in the region given covariates, p(A∣W,A∈𝒜), to estimate how the exposure density profile would change under the restriction.

Alternatively, we might need to make assumptions about what the distribution would look like under the restriction. For example, individuals might aggregate just below the threshold, leading to higher densities near the boundary and lower densities farther from the threshold. However, we do not know this information *a priori*. In practice, this approach would involve building a density estimator that accounts for the restricted set of options for all possible policies, which is computationally infeasible, especially in high-dimensional exposure spaces.

We would then use these individualized densities to calculate the new exposure levels for each person under the restriction, which would require additional data and assumptions about how preferences change. This complexity makes it challenging to model alternative policies without strong assumptions or additional information.

For reasons of interpretability, the frequency of binarizing exposures in the literature (with the aforementioned implicit assumptions), and the ease of using existing tools for estimation, we choose to estimate a policy under the assumption of *relative self-selection*.

In most applications, it is not known *a priori* which region 𝒜 should be set. For example, we may not know how various chemicals or drugs interact and how to set safe limits for all of them. Therefore, 𝒜 itself is, in practice, something that should be estimated to optimize some objective, such as minimizing the expected outcome. However, for the purposes of establishing our theory, we first consider 𝒜 as known and fixed. We then show how we can choose what policy to enact (i.e., choose 𝒜) while also unbiasedly estimating its effect.

We first discuss the assumptions necessary for the statistical quantity estimable from the sample data to have causal interpretations, then we move on to discuss how 𝒜 can be determined.

### 2.4 Identification and causal assumptions

Our target parameter $\psi_{𝒜}$ is defined in terms of the observable distribution and does not represent a causal quantity without additional assumptions. To add a causal interpretation, we define the *causal* ARE as

$$\psi_{𝒜}^{*}=E\left[Y_{𝒜}\right]-E[Y],$$

where $Y_{𝒜}=Y(\tilde{A})$ with $\tilde{A}$ as defined above denoting the counterfactual outcome if exposure A were restricted to 𝒜 while preserving relative per-covariate-stratum preferences. Under the following assumptions, $\psi_{𝒜}^{*}=\psi_{𝒜}$:

1. **Consistency**: If A∈𝒜, then $Y=Y_{𝒜}$.
2. **Conditional Exchangeability (No Unmeasured Confounding)**: $Y_{𝒜}⊥1(A\in 𝒜)|W$.
3. **Positivity**: For all w in the support of $W,\pi_{𝒜}(w)=P(A\in 𝒜|W=w)>0$.

Putting these together:

$$E\left[Y_{𝒜}\right]=E\left[E\left[Y_{𝒜}|W\right]\right]=E\left[\frac{E\left[Y_{𝒜}1_{\left\{A\in 𝒜\right\}}|W\right]}{P(A\in 𝒜|W)}\right]=E\left[E\left[\left.Y\frac{1_{\left\{A\in 𝒜\right\}}}{P(A\in 𝒜|W)}\right|W\right]\right]\;=E[E[Y|T=1,W]].$$

Hence,

$$\psi_{𝒜}^{*}=E\left[Y_{𝒜}\right]-E[Y]=E[E[Y|T=1,W]]-E[Y]=\psi_{𝒜}.$$

Therefore, under these assumptions, the causal attributable regional effect $\psi_{𝒜}^{*}$ is identified by the observable quantity $\psi_{𝒜}$. The remainder of this manuscript will focus on estimating $\psi_{𝒜}$ from the observed data in a manner that is both efficient and robust to model misspecification.

The conditional exchangeability assumption asserts that, within levels of W, the potential outcomes $Y_{𝒜}$ are independent of the exposure A. This implies there are no unmeasured confounders. The positivity assumption ensures that all exposure levels and covariate strata are represented in the data, which is critical for accurate effect estimation.

By satisfying these conditions, we can identify the causal ARE from the observable data. Our focus is on obtaining the observable ARE efficiently without additional assumptions such as linearity. Although our identification assumptions may not always hold in all applications, we aim to eliminate model misspecification bias and minimize random variation.

## 3 Estimating ARE with TMLE

We provide below a high-level summary of the TMLE procedure. For full technical details of the fluctuation model, the offset, and derivation of the influence function-based standard errors, see [11, 12], as these are beyond the scope of the present work.

In the previous sections, we established that the causal ARE is equivalent to the observable ARE $\psi_{𝒜}=E[Q(T=1,W)]-E[Y]$ under standard identification assumptions. Therefore, to estimate it, we need to: (1) create a new binary random variable $T=1_{𝒜}(A)$ and (2) proceed as if we were estimating the average counterfactual effect $\psi_{𝒜}$ from the observational data structure (Y,T,W).

We can adapt techniques for estimating the ATE to construct ARE estimators that offer unbiasedness (assuming no bias from potential violations of identification assumptions) and efficiency. Both Augmented Inverse Probability Weighting (AIPW) and Targeted Maximum Likelihood Estimation (TMLE) are suitable methods when used with machine learning and cross-fitting. Although they often produce similar results, TMLE has been found to perform more efficiently with smaller samples [11, 12], making it a generally preferable approach.

A comprehensive analysis by Li et al. [16] using ten different nutrition intervention studies showed that TMLE, especially in its cross-validated form (CV-TMLE), consistently provided robust and efficient estimates across various realistic simulation scenarios. The study concluded that the additional layer of cross-validation helps avoid unintentional over-fitting of nuisance parameter functionals, leading to more reliable inferences compared to other estimators. This reinforces the advantage of TMLE in practical applications, emphasizing its superior performance in maintaining efficiency and robustness in finite samples. Therefore, for estimating our data-adaptively identified region, we use the CV-TMLE approach.

The TMLE estimator is inspired by the fact that if we knew the true conditional mean Q(T,W)=E[Y∣T,W], we could estimate the ARE with the empirical average $\frac{1}{n}\sum_{i=1}^{n}Q\left(1,W_{i}\right)-Y_{i}$. Of course, we do not know Q, but we can estimate it by regressing the outcome Y on the exposure T and the covariates W. However, in high-dimensional or nonparametric settings, the raw plug-in estimator can exhibit persistent bias that does not vanish asymptotically without additional targeting steps. TMLE corrects this bias by updating the initial regression fits in a way that aligns estimates with the parameter of interest. While we focus on the targeted-learning (TMLE) update, it is worth noting that the one-step estimator – formed by adding the efficient influence function to an initial plug-in fit – offers the same first-order bias reduction; in small samples, however, TMLE’s fluctuation step often yields slightly lower variance [16].

The process is as follows:

1. Use cross-validated ensembles of machine learning algorithms (a “super learner”) to generate estimates of the conditional mean of treatment: $\hat{g}(T=t|W)\approx P(T=t|W)$ (i.e., propensity score) and outcome: $\hat{Q}(T,W)\approx E[Y|T,W]$.
2. Perform a TMLE update to adjust $\hat{Q}$ using the estimated propensity score $\hat{g}$, resulting in a targeted estimate ${\hat{Q}}^{*}$.
3. Compute the plug-in estimate using the targeted model: ${\hat{\psi }}_{𝒜}=\frac{1}{n}\sum_{i=1}^{n}{\hat{Q}}^{*}\left(1,W_{i}\right)-Y_{i}$.

An estimated standard error for ${\hat{\psi }}_{𝒜}$ is given by the empirical variance of the efficient influence function evaluated at the estimated nuisance parameters.

To obtain these estimates, we need only to specify the ensemble of machine learning algorithms used to estimate the propensity and initial outcome regressions $\hat{g}$ and $\hat{Q}$. Theoretical guarantees hold as long as a sufficiently rich library is chosen. To estimate the ARE, we must also specify the region 𝒜 so that we can compute our binary “exposure” variable T. This is the focus of the next section.

## 4 Finding a good exposure region

Our target parameter $\psi_{𝒜}$ depends on the region $𝒜\subseteq R^{m}$ to which we restrict the exposures. When such a region is not already provided (e.g., there are no existing thresholds for a multivariate exposure), it is natural to use the data to discover a region 𝒜 that would optimize population outcomes. Formally, we seek

$${𝒜}^{*}=\underset{𝒜\in 𝒢}{\text{arg}\;\text{min}}\;E\left[Q\left(1,W\right)\right],$$

where 𝒢 denotes the set of all *admissible* regions under consideration. In principle, one might consider all measurable subsets of $R^{m}$, but such a search is computationally infeasible. Instead, we typically restrict 𝒢 to a family of *axis-aligned hyper-rectangles*, ensuring that each 𝒜∈𝒢 can be expressed via thresholds on the exposure coordinates (e.g., $A_{j}\leq \tau_{j}$ for one or more variables). This restriction not only makes the search problem tractable but also improves *interpretability* of the resulting threshold-based policy.

When searching over these regions, we also want to maintain the positivity assumption, which requires that P(A∈𝒜∣W=w) is bounded away from 0 and 1 for all covariate values w in the support of W. This helps avoid instability or infinite variance in the estimates.

Even with these axis-aligned constraints, estimating the “oracle” region ${𝒜}^{*}$ efficiently is highly non-trivial. In principle, one would have to compute plug-in estimates ${\hat{\psi }}_{𝒜}$ for every 𝒜∈𝒢, which quickly becomes infeasible in moderate to large dimensions. To strike a balance between flexibility and efficiency, we propose a greedy *recursive partitioning* algorithm that builds a piecewise-constant approximation of the optimal region. At each step, the algorithm only pursues new splits if the estimated conditional probability of being in the proposed subregion remains bounded above ϵ>0 and below 1-ϵ, thereby enforcing the positivity requirement.

Moreover, we note that additional constraints can be imposed to prevent degenerate cases (e.g., single-point solutions when μ(a,w) has a unique minimum). In the univariate case, for example, $a^{2}$ attains a global minimum at a=0, so ${𝒜}^{*}=\{0\}$ is not amenable to standard causal inference due to zero support. Our positivity restrictions circumvent such extremes, effectively ruling out set(s) 𝒜 that occur with probability zero or nearly zero.

The next subsection details how we implement this recursive partitioning scheme to approximate ${𝒜}^{*}$ from the observed data.

### 4.1 Targeted decision trees

We introduce a novel decision tree algorithm developed to approximate the oracle parameter. This parameter, denoted as

$${𝒜}^{*}=\underset{𝒜\in 𝒢}{\text{arg}\;\text{min}}\;E[E[Y|T=1,W]],$$

represents the region 𝒜 that minimizes the expected outcome for a given set of covariates W. Our approach is tailored to meet several key criteria: computational efficiency, interpretability, and implicit regularization to avoid overly granular and statistically insignificant regions.

#### 4.1.1 Implicit regularization via split criteria

In this context, “implicit regularization” refers to the fact that new splits in the decision tree are only made when certain criteria are met: (*i*) a minimum node size is enforced (so that each child node contains sufficient observations), (*ii*) positivity constraints discard potential splits with near-zero probability of receiving treatment (T=1), and (*iii*) a p-value threshold ensures that each proposed split must yield a statistically meaningful improvement in the targeted parameter of interest. Consequently, the algorithm cannot continue subdividing the data unless it finds evidence of a substantial change in estimated average outcome. Thus, *without* requiring additional pruning or penalization, the tree is prevented from growing excessively and carving out tiny regions that might overfit the data.

#### 4.1.2 Partitioning procedure

The core of our method is a regression tree that optimizes a simple plug-in estimator for the policy effect E[Y∣T=1,W] of a given axis-aligned rectangular region. This is based on using Random Forest or a similar straightforward model for nuisance parameter estimation paired with targeted learning. Our decision tree is constructed to search for the minimum (or maximum) region using this plug-in estimator, greedily evaluating splits in the exposure variables A and selecting the region that minimizes (or maximizes) the estimated average outcome in the newly formed regions. In Section 5.1, we detail how statistical tests on the estimated causal quantity filter each split, naturally inhibiting excessive branching of the tree and promoting transparent, interpretable thresholds in multivariate exposure spaces.

### 4.2 Positivity and practical diagnostics

Positivity is a fundamental requirement for identification of causal effects, ensuring that every relevant subregion 𝒜 occurs with nontrivial probability across the covariate support. In practice, certain subpopulations may rarely (or never) appear with A∈𝒜, leading to sparse data and large variance if one attempts estimation there. Our algorithm guards against such issues by discarding (or merging) any partitions that yield near-zero probabilities for some W stratum. Specifically, if the empirical probability ${\hat{\pi }}_{i}=P\left(T=1|W_{i}\right)$ dips below a user-specified ϵ>0 in that sub-node, we refrain from splitting on that cutpoint.

To implement this positivity check, we monitor sample sizes and empirical probabilities in each node as the tree grows. If a node’s effective cell counts become very small in some covariate strata, we deem that region unfit for stable estimation. Although this may lead to a slightly less aggressive search for the “best” threshold, it ensures that the final subregion 𝒜 remains well-supported by the data. From a causal standpoint, if certain individuals in W never fall into 𝒜, no statistical procedure can reliably learn the counterfactual outcome there.

Empirically, we recommend choosing ϵ in the range [0.001, 0.05], depending on sample size and whether more permissive or more conservative splits are appropriate. If ϵ is too large, the tree may under-split; if ϵ is too small, it may keep pursuing splits in highly sparse regions, risking unstable or extrapolative estimates. We also encourage a minimum node-size restriction (e.g. 10, 20, or more) to reinforce these positivity checks. Together, these diagnostics help maintain robustness and interpretability in real data settings.

### 4.3 Algorithm overview

Algorithm 1 outlines our recursive partitioning procedure. Below, we summarize its five main phases:

#### 4.3.1 Partitioning criterion phase

For each exposure variable $A_{j}$, the algorithm considers a set of potential thresholds s, taken either from *unique observed* values (possibly rounded or binned) or from predefined domain knowledge (e.g. official safety cut points). In other words, we enumerate all candidate splits $A_{j}\leq s$ for all j.

#### 4.3.2 Debiasing with TMLE

For each candidate split, we define $T_{i}=1\left\{A_{j}\leq s\right\}$ and use machine learning to obtain initial estimates of ${\hat{\theta }}_{\text{left}}$ and ${\hat{\theta }}_{\text{right}}$ (i.e., the mean outcome for T=1 vs. T=0). We then apply Targeted Maximum Likelihood Estimation (TMLE) to *update* these fits, removing asymptotic bias in the conditional mean estimates.

#### 4.3.3 Significance testing

Next, we compute the difference in mean outcome between each child node and the parent node, along with its p-value (using the TMLE influence function). If the p-value lies below a user-specified significance level α, we consider that split valid. This step ensures that any further partitioning reflects a meaningful shift in the causal parameter of interest. This helps find meaningful regions in finite samples but becomes less stringent as sample size increases and significance is easier to attain.

#### 4.3.4 Optimal split selection

Among all valid splits, we select the one yielding the largest improvement in outcome in the desired direction (minimizing or maximizing). If no valid splits pass the positivity check (i.e. ${\hat{\pi }}_{i}$ outside [ϵ,1-ϵ]) or fail significance, the node becomes a leaf.

#### 4.3.5 Recursive partitioning

We continue splitting each child node until reaching the maximum tree depth $d_{\text{max}}$ or until no further splits improve the outcome. Ultimately, each path from the root to a leaf defines a subregion in the exposure space whose average outcome is estimated under relative self-selection.

**Algorithm 1:** Recursive Partitioning Decision Tree with TMLE.

1: **Input:** Data D, exposures A, covariates W, outcome Y, max depth $d_{\text{max}}$, objective ∈ {min, max}, positivity threshold ϵ,p-value threshold α
2: **Procedure** BuildTree(D, depth, parentMean):
1. If $\text{depth}\geq d_{\text{max}}$ **return** leaf with mean = parentMean.
2. For each exposure $A_{j}$ and each candidate threshold s:
(a) Define $T_{i}=1\{A_{j}\leq s\}$ and estimate $\hat{\pi }=P(T_{i}=1|W_{i})$.
(b) If any ${\hat{\pi }}_{i}<\epsilon$ or >1-ϵ, **skip** (positivity fails).
(c) Fit TMLE for mean outcome in T=1 vs. T=0, yielding ${\hat{\theta }}_{\text{left}}$ and ${\hat{\theta }}_{\text{right}}$.
(d) Compute p-values for $\Delta_{\text{left}}={\hat{\theta }}_{\text{left}}$ − parentMean and similarly for $\Delta_{\text{right}}$.
(e) If p-value <α **and** child node’s mean is improved (lower if objective = “min”, higher if = “max”), track it.
3. If no valid splits found, **return** leaf with mean = parentMean.
4. Choose the best valid split (lowest/highest mean). Recursively call BuildTree on each resulting child node, incrementing depth, and passing in its parentMean.
3. **Initialize**: BuildTree($D,0,\frac{1}{n}\sum_{i}Y_{i}$)

### 4.4 Choosing the maximum tree depth *d*_max_

Depth controls a three-way trade-off: (i) *interpretability* – every extra split adds another Boolean clause; (ii) *sampling variability* – smaller leaves inflate variance and bias; (iii) *empirical positivity* – deep trees more easily violate the lower-bound constraint.

#### Practical rule of thumb.

For mixtures with ≤10 exposures a depth of 2–3 almost always exhausts the signal while keeping rules readable. If m>10, begin with $d_{\text{max}}=1$ and increase only when the *cross-validated* risk (Section 6) improves. Analysts needing additional assurance may grid-search $d_{\text{max}}\in \{1,2,3\}$ in an outer CV loop; the computational cost is modest because trees remain shallow.

#### Consistency check.

Large fold-to-fold disagreement in the selected interaction pattern usually indicates the depth is too large (or ε too small) for the sample size; reducing $d_{\text{max}}$ by one typically restores stability.

## 5 Positioning our causal decision tree in the literature

### 5.1 Significance-filtering splitting criterion

In classical decision tree frameworks (e.g., CART; [17]), splits typically aim to optimize predictive performance – such as minimizing within-node variance – without explicit consideration of a *causal* parameter. By contrast, our approach centers on identifying threshold-based subregions that meaningfully shift the *policy-relevant* attributable regional effect (ARE). To accomplish this, we evaluate each proposed split in terms of the child node’s TMLE-based difference from the parent node, then apply a p-value threshold to ensure the difference is statistically significant. This procedure is reminiscent of the test-based splitting used in “conditional inference trees” [18], though we combine it with a targeted learning framework tailored to causal parameters rather than predictive error. Because our test statistic compares directly against the parent node’s estimate of the *same* semiparametric quantity, it offers a principled basis for discarding weak or spurious splits, thus guiding the tree expansion toward subregions that truly deviate in the estimated intervention effect. In effect, we only partition further when robust evidence suggests a given subregion alters the average outcome in a manner consistent with our *oracle* parameter of interest. This safeguards against overfitting and preserves interpretability, as each split directly reflects a significant change in the policy-based target rather than mere noise in the predictive fit.

### 5.2 Relation to honest trees and causal forests

The “honest” tree framework of Athey and Imbens [19] and the causal forest approach of Wager and Athey [20] both rely on rigorous sample-splitting to mitigate bias when estimating heterogeneous treatment effects in W-space. In particular, honest trees separate the data used to build (or select) partitions from the data used to estimate treatment effects in each leaf. Our cross-validated procedure likewise prevents reusing the same observations for both partition discovery and final effect estimation.

Despite these conceptual parallels, there are two key distinctions in our method:

1. **Focus on Exposure Thresholds.** Rather than identifying subpopulations of W that exhibit differential responses, we search for threshold-based constraints on A that globally optimize a population-level objective. This shift reflects our policy-oriented parameter: we seek a single region 𝒜 that would hypothetically improve outcomes if everyone were forced to lie within it, subject to positivity. By contrast, honest trees and causal forests typically aim to find subgroups in W that have distinct conditional average treatment effects for a binary exposure.
2. **TMLE-Driven Significance.** We explicitly employ TMLE (and an associated significance filter) at each split to assess changes in our policy-based causal quantity. In causal forests, each leaf often provides a local average treatment effect, whereas our method seeks to discover a subregion 𝒜⊆A for a single intervention that (globally) minimizes or maximizes the outcome. Although this divergence in target parameters alters the splitting logic, both approaches share the concept of *honest* partitioning: the data used to define the partition must be distinct from the data used to estimate the effect in that partition, mitigating overfitting bias.

## 6 K-fold cross-estimation

As discussed, the identification of interaction regions and the estimation of ARE requires a data partitioning strategy that mitigates bias. We employ a K-fold cross-estimation technique that ensures the asymptotic properties of our estimators without reliance on additional assumptions. This involves dividing the data set into complementary estimation ($P_{n_{k}}$) and parameter-generating ($P_{n_{-k}}$) samples, the latter being utilized to derive exposure regions and train nuisance parameters essential for the TMLE update. For each fold, we determine the exposure thresholds using $P_{n_{-k}}$ and, with the same sample, train our nuisance estimators $\hat{g}$ and $\hat{Q}$. Subsequently, these estimators are applied to $P_{n_{k}}$ to obtain unbiased ARE estimates within the fold.

### 6.1 Pooled TMLE

Upon completion of the cross-estimation procedure, a pooled TMLE update provides a summary measure of the oracle region target parameter across folds. Specifically, we stack the predictions for each nuisance parameter from the estimation samples and run a pooled TMLE update on the cumulative initial estimates. The resulting average is then used for parameter estimation.

Our substitution estimator, denoted by ${\hat{Q}}_{n}$, approximates the true conditional mean $Q_{0}$ by plugging in the empirical distribution for each observation. The estimator is operationalized via a Super Learner algorithm followed by TMLE, and its cross-validated variant is expressed as

$${\hat{\Psi }}_{\text{CV}}=\frac{1}{K}\sum_{k=1}^{K}\{\frac{1}{n_{k}}\sum_{i\in P_{n_{k}}}{\hat{Q}}_{n_{-k}}^{*}\left(1,W_{i}\right)-Y_{i}\},$$

where ${\hat{Q}}_{n_{-k}}^{*}$ is the TMLE-updated expectation of the outcome if everyone were exposed to levels within the data-adaptively determined region. The cross-estimation approach not only utilizes the full dataset for variance estimation, resulting in tighter confidence intervals, but also enables the derivation of fold-specific and pooled estimates.

That is, this pooled parameter represents the ARE for the determined maximizing/minimizing region in the exposure space. Of course, the actual regions determined in folds can vary, and thus this acts as an omnibus test, which can be used to evaluate fold-specific results. A significant pooled TMLE ARE for the oracle region can be interpreted as “there is a region in the exposure space where the expected outcome is significantly different compared to the average outcome”. The analyst can then evaluate the fold-specific results to determine what regions make up this oracle estimate. Of course, interpretability of the pooled oracle estimate is reliant on regions in the same sets of exposures that are found across the folds. Otherwise, this pooled estimate allows the analyst to understand if there is generally “signal” in the data for a region or set of regions that collectively differ in expected outcomes compared to the average. This dual presentation allows for the evaluation of variability across folds, offering insight into the stability of the pooled ARE. In particular, when exposure regions 𝒜 exhibit high variability between folds, fold-specific results provide an essential interpretive counterbalance to the pooled estimates.

## 7 Theoretical properties of the estimator

Thus far, we have introduced the Attributable Regional Effect (ARE) for a subregion $𝒜\subseteq R^{m}$ of the exposure space, emphasizing that 𝒜 is *unknown a priori* but can be *discovered* from the data by threshold-based partitioning (Algorithm 1). We then employ cross-validated TMLE to estimate and infer the causal impact of restricting exposures to that region. This section clarifies *why* such a data-adaptive procedure, which uses the same dataset to both find and evaluate a subregion, remains valid under mild assumptions. We also discuss the difference between a *globally optimal* partition search and a more scalable *greedy* recursive strategy.

### 7.1 Data-adaptive parameters and cross-validated TMLE

Classical semiparametric inference on treatment effects (e.g., the ATE) focuses on fixed target parameters. Here, by contrast, the parameter

$$\psi_{𝒜}=\Psi \left(P_{0},𝒜\right)$$

depends on a region 𝒜 that is identified by the data for the purpose of minimizing (or maximizing) the population mean outcome. Specifically, we aim to approximate the “oracle” region

$${𝒜}^{*}=\underset{𝒜\subseteq R^{m}}{\text{arg}\;\text{min}}\;\Psi \left(P_{0},𝒜\right),$$

making $\left({𝒜}^{*},\psi_{{𝒜}^{*}}\right)$ a *data-adaptive* target parameter [14]. When ${𝒜}^{*}$ is learned with the *same* sample used for inference, naive estimators can severely overfit, compromising validity.

To address this, we adopt a K-fold cross-validation (CV) approach (Section 6), which splits the dataset into K folds. For each fold k, we use the *training sample* $P_{n_{-k}}$ (everything except fold k) to (i) estimate the nuisance parameters ${\hat{Q}}_{n_{-k}},{\hat{g}}_{n_{-k}}$, *and* (ii) discover a region ${\hat{𝒜}}_{n_{-k}}$. We then apply that region and those fits to the held-out data $P_{n_{k}}$ to get an unbiased estimate of $\psi_{𝒜}$. Averaging these fold-specific estimates yields the *cross-validated TMLE*, which is asymptotically unbiased for $\psi_{{𝒜}^{*}}$ under conditions outlined below [11, 14, 21].

### 7.2 Global versus greedy partitioning

While cross-validation mitigates overfitting bias, the question remains how to search for the optimal region ${𝒜}^{*}$ within the exposure space. In principle, one might consider every axis-aligned subregion of $R^{m}$ – an intractable set in most settings. We therefore highlight two main strategies:

#### 7.2.1 Global search (shallow depth)

If the decision tree depth $d_{\text{max}}$ is small and the exposures are either low-dimensional, discretized, or binned based on some rounding, one can *exhaustively enumerate* potential splits. Concretely, each exposure coordinate $A_{j}$ is restricted to a set of candidate thresholds (e.g., unique observed values), and we systematically form all hyperrectangles in up to $d_{\text{max}}$ splits. Evaluating each candidate 𝒜 via a plug-in or TMLE estimate of $\Psi \left(P_{n_{-k}},𝒜\right)$ yields an *empirical* minimizer, which converges to the true ${𝒜}^{*}$ as n→∞ under standard assumptions (positivity, consistent nuisance estimation, no unmeasured confounders). Recent results show that such “optimal tree” searches can be formulated as mixed-integer programs [22–24], guaranteeing a global solution for shallow depths. However, the complexity of enumerating subregions can grow exponentially in m and $d_{\text{max}}$, making a *greedy* approach more practical in higher dimensions.

#### 7.2.2 Greedy recursive partitioning

Algorithm 1 instead takes a *CART-like* strategy [17]: at each node, it selects the split that yields the largest local improvement in outcome (*e.g.*, lowers the conditional mean if minimizing), subject to positivity and statistical significance constraints. This strategy is *computationally efficient* but can find only a *local* optimum if there are multiple partitions with similar risk. In practice, when the threshold signal is sufficiently strong, greedy splitting (in small depths) often provides an effective approximation to the global minimizer, especially when combined with cross-fitting or “honest” sample splits [19]. Indeed, we observe empirically in Sections 8 and 9 that the greedy procedure typically recovers meaningful threshold structures.

### 7.3 Optional exhaustive search for the optimal region

While the aforementioned decision tree algorithm follows a *greedy* strategy to quickly locate a high-performing subregion, it may identify only a *locally* optimal path of splits at each node. In some settings, especially with few exposures or a moderate tree depth limit, a more global search may be computationally tractable. To this end, we include an *exhaustive* search option in CVtreeMLE, designed to guarantee finding the *globally* best terminal leaf (i.e., the lowest- or highest-mean leaf, depending on min_max) among all valid partitions up to a specified depth.

#### 7.3.1 Algorithmic overview

When exhaustive = TRUE, each node considers *every* candidate exposure threshold meeting the positivity and p-value criteria described earlier (Sections 4–5.1). Rather than picking just the single top-scoring split (as in the greedy approach), the exhaustive mode:

1. **Branches** on all thresholds that both pass the significance test and improve the parent node’s mean outcome in the desired direction (minimum or maximum).
2. Recursively applies this branching procedure to each child node, up to the user-specified max_depth.
3. Collects all resulting *terminal leaves* from every branch once no further valid splits can be made.
4. Selects the single best leaf among these, measured by the smallest or largest estimated mean outcome (depending on min_max).

Hence, the algorithm produces a globally optimal subregion in the sense that no other leaf in the entire search space (up to max_depth) has a more extreme mean.

#### 7.3.2 Computational considerations

Exhaustively branching on every valid threshold can expand the search tree exponentially, causing runtime to increase rapidly with the number of exposure variables, candidate split points, or allowed tree depth. In practice, exhaustive mode is most appropriate when:

- The dimensionality of the exposure space is relatively small, or the exposures are binned/coarsened sufficiently.
- The desired depth (i.e., the number of sequential splits) is modest, such that the total number of candidate subregions remains manageable.
- The analyst wishes to ensure no globally better region is overlooked by a purely local (greedy) strategy.

#### 7.3.3 Practical implications

In higher-dimensional contexts or when each exposure has many finely spaced potential cutpoints, the branching factor becomes large. Consequently, the greedy search described in Section 4 typically remains far more computationally efficient and, under mild assumptions, converges to an equivalent solution in large samples (cf. Section 7.2). By contrast, the exhaustive procedure can locate the global optimum whenever computational resources permit a full enumeration of possible splits and sub-splits. This dual approach – greedy versus exhaustive – thus offers users the flexibility to balance interpretability, guaranteed optimality, and runtime constraints, depending on the structure and scale of their mixtures data.

### 7.4 Consistency and convergence

Having defined our data-adaptive procedure for discovering a subregion ${\overset{ˆ}{𝒜}}_{n}$, we now present theoretical conditions ensuring that it converges to the “oracle” region ${𝒜}^{*}\subseteq 𝒢$ that optimizes (minimizes or maximizes) the target parameter $\Psi \left(P_{0},𝒜\right)$. Specifically, suppose there exists a true region

$${𝒜}^{*}=\underset{𝒜\in 𝒢}{\text{arg}\;\text{min}}\;\Psi \left(P_{0},𝒜\right),$$

and we estimate it via ${\overset{ˆ}{𝒜}}_{n}$ and evaluate ${\hat{\Psi }}_{n}=\Psi \left(P_{n},{\overset{ˆ}{𝒜}}_{n}\right)$. Below, we outline high-level assumptions ensuring $\left({\overset{ˆ}{𝒜}}_{n},{\hat{\Psi }}_{n}\right)\to \left({𝒜}^{*},\Psi \left(P_{0},{𝒜}^{*}\right)\right)$ in probability.

**Theorem 1:** (Consistency and Convergence of Data-Adaptive Regions). Let $\left\{\Psi_{\theta }:\theta \in \Theta \right\}$ be a family of pathwisedifferentiable target parameters mapping P to real values, each with canonical gradient $\phi_{\theta }$. Assume (Θ,d) is a metric space, and for each P we write $\psi_{\theta }=\Psi_{\theta }(P)$. Let $\psi_{\theta ,n}={\hat{\Psi }}_{\theta }\left(P_{n}\right)$ be estimators of $\psi_{\theta }$, where $P_{n}$ is the empirical distribution of an i.i.d. sample from P. Suppose:

1. **Separated Maximum:** There is a unique $\theta_{0}\in \Theta$ such that
$$\underset{\theta \in \Theta }{\text{max}}\;\psi_{\theta }=\psi_{\theta_{0}},\;\text{and}\;\underset{\left\{\theta :d\left(\theta ,\theta_{0}\right)\geq \epsilon \right\}}{\text{sup}}\psi_{\theta }<\psi_{\theta_{0}}\;\text{for}\;\text{all}\;\epsilon >0.$$
2. **Glivenko-Cantelli Class of Gradients:** $\left\{\phi_{\theta }:\theta \in \Theta \right\}$ is Glivenko-Cantelli so that ${\text{sup}}_{\theta \in \Theta }\left|\left(P_{n}-P\right)\phi_{\theta }\right|=o_{P}(1)$.
3. **Uniformly Vanishing Remainders:** The estimators $\psi_{\theta ,n}$ are asymptotically linear with influence functions $\phi_{\theta }$, satisfying
$$\underset{\theta \in \Theta }{\text{sup}}\left|\left(\psi_{\theta ,n}-\psi_{\theta }\right)-\left(P_{n}-P\right)\phi_{\theta }\right|=o_{P}\left(1\right).$$
  Let $\theta_{n}$ be the data-adaptive maximizer of $\theta \mapsto \psi_{\theta ,n}$. Then $\theta_{n}\to^{p}\theta_{0}$.

**Proof Sketch**. Because $\theta_{0}$ is a strict arg max, it suffices (by van der Vaart’s Argmax Theorem [25, Theorem 5.7]) to show that ${\text{sup}}_{\theta \in \Theta }\left|\psi_{\theta ,n}-\psi_{\theta }\right|\to^{p}0$. We decompose

$$\psi_{\theta ,n}-\psi_{\theta }=\left(P_{n}-P\right)\phi_{\theta }+\left\{\left(\psi_{\theta ,n}-\psi_{\theta }\right)-\left(P_{n}-P\right)\phi_{\theta }\right\}.$$

Label the curly-bracketed difference as $R_{\theta }$. Then

$$\underset{\theta \in \Theta }{\text{sup}}\left|\psi_{\theta ,n}-\psi_{\theta }\right|\leq \underset{\theta \in \Theta }{\text{sup}}\left|\left(P_{n}-P\right)\phi_{\theta }\right|+\underset{\theta \in \Theta }{\text{sup}}\left|R_{\theta }\right|.$$

By assumption (ii), ${\text{sup}}_{\theta }\left|\left(P_{n}-P\right)\phi_{\theta }\right|=o_{P}(1)$. By assumption (iii), ${\text{sup}}_{\theta }\left|R_{\theta }\right|=o_{P}(1)$. Hence the entire supremum vanishes in probability, ensuring arg ${\text{max}}_{\theta }\psi_{\theta ,n}\to \theta_{0}$. □ □

This argument is theoretical and does not directly apply to our procedure but it sets up some intuition that motivates our choices in finding a target parameter using recursive partitioning. For one, it shows that when a region gives the empirical maximum estimate among all considered regions (and other regularity conditions hold) then selecting it eventually leads to convergence to the appropriate region. This requires us to take the global empirical maximum over regions, which we approximate with a greedy algorithm: this is a trade-off with computational speed.

In terms of regularity conditions, the proof first requires a separated maximum. This condition is more likely to attain when we consider a class of regions that are constrained-this is one reason that we use shallow trees. Another reason is that the Glivenko-Cantelli condition is more easily satisfied when the index set of regions is small. Lastly, the proof requires *uniformly* vanishing remainders. It is difficult to verify this condition but again keeping the index set small helps us accomplish this.

As our simulations (Section 8) show, even a greedy strategy converges well when signal strength is moderate or strong and sample sizes are reasonably large. The real-data applications (Section 9) illustrate that CVtreeMLE can uncover meaningful threshold structures in more complex settings. Thus, both the theoretical guarantees and empirical results affirm the feasibility and reliability of our data-adaptive approach.

## 8 Simulations

In this section, we demonstrate using simulations that our approach identifies the correct exposure region that maximizes/minimizes the conditional mean and estimates the correct ARE target parameter built into a data-generating process (DGP) for this region. Henceforth we refer to our method as CVtreeMLE.

### 8.1 Two-dimensional exposure simulations

We created a squared dose-response relationship between two exposure variables where an interaction occurs between the exposures when each meets a particular threshold value. Specific outcome values were generated for each subspace of the mixture based on split points ${𝒜}_{d}$, but there exists a region with the maximum outcome (the truth that we want). The goal is to determine whether our adaptive target parameter is targeting the region that maximizes/minimizes the conditional mean outcome for the given sample and to evaluate how CVtreeMLE approaches this desired oracle parameter (the true ARE for this region) as the sample size increases.

This DGP has the following characteristics: O=(W,A,Y). W are three baseline covariates, $W_{1}\sim 𝒩(\mu =37,\sigma =3),W_{2}\sim 𝒩(\mu =20,\sigma =1),W_{3}\sim \text{Bernoulli}(0.5)$. These distributions and values were chosen to represent a study with covariates for age, BMI, and sex.

Our generated exposures were created to represent two chemical exposures quantized into five discrete levels. We sample observations into a 5 × 5 grid based on combinations of two discrete exposure levels with values 1–5. We define a conditional categorical distribution $P\left\{\left(A_{1},A_{2}\right)=\left(a_{1},a_{2}\right)|W=w\right\}$ and sample from it. The probabilities are defined using a multinomial logistic model with coefficients drawn from normal distributions.

We assign an outcome to each of these regions based on the main effects and interactions between exposures:

$$Y=0.2A_{1}^{2}+0.5A_{1}A_{2}+0.5A_{2}^{2}+0.2W_{1}+0.4W_{3}+\varepsilon ,$$

where ε~𝒩(0,0.1). This indicates that there is a slightly weaker squared effect for $A_{2}$ relative to $A_{1}$ and a strong interaction between the exposures and confounding due to $W_{1}$ and $W_{3}$.

#### 8.1.1 Computing ground truth

In our discrete-exposure simulation, each exposure $A_{j}$ takes values in a finite set (e.g., {1, 2, 3, 4, 5}). This allows us to *enumerate every possible subregion* $𝒜\subseteq R^{m}$ by selecting subsets of these discrete levels for each exposure coordinate. For each candidate subregion 𝒜, we can compute the *adjusted* (or *counterfactual*) expected outcome conditioned on W=w as

$$Q_{𝒜}\left(1,w\right)=\frac{\sum_{a\in 𝒜}\mu_{0}\left(a,w\right)p_{A|W}\left(a|w\right)}{\sum_{a\in 𝒜}p_{A|W}\left(a|w\right)},$$

where $\mu_{0}(a,w)=E[Y|A=a,W=w]$ is the true (known) conditional mean in our simulation. By integrating out W under the true data-generating distribution $p_{\text{W}}$, we obtain the population-level outcome under an intervention forcing A∈𝒜:

$$E\left[Q_{𝒜}(1,W)\right]=\int Q_{𝒜}(1,w)p_{W}(w)\text{d}w.$$

We define the attributable regional effect (ARE) of 𝒜 as

$$\text{ARE}(𝒜)=E\left[Q_{𝒜}(1,W)\right]-E[Y].$$

Because the exposure space here is discrete and low-dimensional, we can compute ARE(𝒜) for all candidate 𝒜 and select the oracle region ${𝒜}^{*}$ that *maximizes* the adjusted outcome, in this case, the maximizing region was found to be when both exposures are equal to 5. The true ARE for this region was found to be 37.1.

Importantly, in the full population distribution $P_{0}$, *positivity* is well satisfied for every w and each candidate 𝒜. In a finite sample of size n, however, some subregions may become sparse (i.e., nearly zero observations for certain w-strata), violating empirical positivity. Our tree-based algorithm thus may discard those subregions (or shrink them toward larger sets) to maintain stable estimation. Consequently, the discovered subregion in a small sample might differ slightly from the true ${𝒜}^{*}$, but as n increases, the empirical distribution converges to $P_{0}$ and our data-adaptive procedure recovers the oracle region under standard conditions (see Section 7.2). Thus, we expect to see higher bias and lower coverage when comparing our estimates to ground truth for lower sample sizes which converge to the truth when n is large. We also expect to see that the data-adaptive parameter (comparing our estimate to the true estimand when the discovered rule is applied to $P_{0}$), should have good coverage across all sample sizes.

### 8.2 Simulation study with continuous exposures

In this simulation, we generate a large dataset (n=100,000) in order to (i) create realistic continuous, correlated exposures that driven by baseline covariates and (ii) enforce a known threshold-based effect on the outcome. Specifically:

1. **Baseline Covariates**. We first sample three baseline covariates for each individual:
$$W_{1}\sim N(37,3),\;W_{2}\sim N(20,1),\;W_{3}\sim \text{Bernoulli}(0.5).$$
  These represent possible confounding variables typically seen in biomedical contexts.
2. **Exposure Generation**. We next create two correlated *continuous exposures* $A_{1}$ and $A_{2}$, each dependent on the covariates ($W_{1},W_{2},W_{3}$). Formally, we specify
$$E\left[A_{1}|W\right]=\alpha_{0}^{\left(1\right)}+\alpha_{1}^{\left(1\right)}\cdot W_{1}+\alpha_{2}^{\left(1\right)}\cdot W_{2}+\alpha_{3}^{\left(1\right)}\cdot W_{3},$$
$$E\left[A_{2}|W\right]=\alpha_{0}^{\left(2\right)}+\alpha_{1}^{\left(2\right)}\cdot W_{1}+\alpha_{2}^{\left(2\right)}\cdot W_{2}+\alpha_{3}^{\left(2\right)}\cdot W_{3},$$
  and we add a correlation structure via a covariance matrix Σ, set to have correlation 0.5 between $A_{1}$ and $A_{2}$. Concretely, we draw each ($A_{1},A_{2}$) using
$$\left(A_{1},A_{2}\right)\sim \text{MVN}\left(\mu_{\left(A_{1},A_{2}\right)}\left(W\right),\Sigma \right),$$
  with $\Sigma =\left(\begin{array}{cc} 1 & 0.5\\ 0.5 & 1 \end{array}\right)$. This enforces that $A_{1}$ and $A_{2}$ are *continuous*, correlated random variables, *and* confounded by the baseline covariates.
3. **Rounding**. To avoid an explosively large number of unique split points in the decision tree, we *round* $A_{1}$ and $A_{2}$ to one decimal place. This minor binning maintains primarily continuous behavior while still keeping the candidate threshold space tractable for the partitioning algorithm.
4. **Outcome Model with Threshold Effects**. Finally, we define the outcome Y such that there is a known threshold-based signal in $A_{1}$ and $A_{2}$, together with direct confounding effects from $W_{1}$ and $W_{2}$. Specifically,
$$Y=3\cdot 1\left\{A_{1}>0.5\right\}+2\cdot 1\left\{A_{2}<-0.2\right\}+0.2W_{1}+0.4W_{2}+\epsilon ,$$
  where ϵ~N(0,0.1) is small Gaussian noise. In this way, $A_{1}>0.5$ and $A_{2}<-0.2$ define the *true* region of interest for which the outcome is maximized. Moreover, $W_{1}$ and $W_{2}$ jointly confound the exposures and outcome because they appear both in the exposures’ linear predictors and directly in Y. Although only $W_{1}$ and $W_{2}$ enter the outcome model, $W_{3}$ still predicts the exposures. Including a non-confounding covariate lets us verify that CV-TMLE remains unbiased even when irrelevant variables are supplied; omitting $W_{3}$ would give identical large-sample results.

This data-generating process thus yields a *continuous exposure* scenario, with built-in correlation and confounding, and a latent threshold-based “oracle” region ($A_{1}>0.5,A_{2}<-0.2$). In the subsequent analyses, we repeatedly sample smaller n-sized subsets (200, 500, 1,000, 2,000) from this large dataset, apply our recursive decision tree and targeted estimation procedures, and evaluate how effectively our method identifies the correct threshold-based region in a realistic continuous-exposure setting.

### 8.3 Null simulation: outcome independent of exposures

To assess how our proposed method behaves in the absence of any exposure effect, we conducted a simulation where the outcome is strictly independent of the exposures. Specifically, we generated $P_{0}$ of size n=100,000 from the following data-generating process (DGP):

1. **Covariates**. For each individual i∈{1,…,n}, we sample baseline covariates, ($W_{i,1},W_{i,2},W_{i,3}$) where $W_{1}\sim N(50,10)$, $W_{2}\sim N(25,3)$, and $W_{3}\sim \text{Bernoulli}(0.5)$.
2. **Exposures**. We then generate *continuous*, correlated exposures ($A_{1},A_{2}$) that may depend on the covariates W, using a linear mean function plus a correlation structure. Concretely, we let
$$\mu_{1}\left(W\right)=0.3\;W_{1}+0.1\;W_{2}+0.2\;W_{3},\;\mu_{2}\left(W\right)=-0.1\;W_{1}+0.2\;W_{2},$$
  and draw ($A_{1},A_{2}$) from a bivariate normal with mean ($\mu_{1}(W)$, $\mu_{2}(W)$) and covariance
$$\Sigma =\left(\begin{array}{cc} 1 & 0.5\\ 0.5 & 1 \end{array}\right).$$
  This ensures some correlation between $A_{1}$ and $A_{2}$, as well as confounding structure whereby W influences $A_{1}$ and $A_{2}$. We round each exposure to one decimal place to keep the number of potential split points finite.
3. **Outcome (Null)**. Crucially, the outcome Y does *not* depend on $\left(A_{1},A_{2}\right)$. Instead, we define
$$Y=\varepsilon ,\;\varepsilon \sim N\left(0.2\cdot W_{3},5^{2}\right),$$
  so that $W_{3}$ has a mild effect on Y but there is *no direct or indirect relationship* between Y and ($A_{1},A_{2}$). Thus, any attempt to discover an “optimal” exposure region should fail except by chance.

Because Y is generated independently of A, the covariates $W_{1}$ and $W_{2}$ are *not* confounders in this design; the estimator would remain consistent were they omitted. We retain them to mimic a realistic analyst who is uncertain which baseline factors are relevant. Once the population is generated, we repeatedly draw smaller samples of size n∈{200,500,1,000,2,000,5,000} to simulate different data set sizes and run our CVtreeMLE procedure on each subsample. We set a nominal p-value threshold (e.g., α=0.05) as part of the splitting rule: splits are only retained if they yield a statistically significant improvement in the estimated attributable regional effect (ARE). Under this null DGP, any apparent “benefit” from partitioning the exposure space should be purely due to random noise; hence, we expect most partitions to fail the significance test. Of note, if any one fold returns no region detected the CVtreeMLE algorithm returns “no consistent region found”, this is because if a fold has no region detected then no pooling effects can be estimated. We run this simulation with 2 fold CV and therefore expect a spurious region found in all folds 0.025 % of the time, or basically 0.

#### 8.3.1 Metrics

For each simulated data set, we record:

- Whether the algorithm *selects a non-empty* region ${𝒜}^{*}$ (despite the true effect being zero).
- The fraction of times the CVtreeMLE procedure reports a statistically significant region across all simulation replicates (Type I error rate).
- The average *estimated* ARE, ${\hat{\psi }}_{𝒜}$, for any discovered region, which should be close to zero if the method is well-calibrated.
- Coverage of the 95 % confidence interval around zero, if a region is discovered.

#### 8.3.2 Interpretation

Because our algorithm only deems a region “found” if *every* cross-validation fold discovers and confirms a significant split, the *overall* chance of detecting a spurious region in a strict null scenario is approximately $\alpha^{K}$, rather than α. For example, with ten folds (K=10) and α=0.05, that rate would be $0.05^{10}\approx 10^{-14}$, which is far below 5 %. Consequently, when there is truly no exposure effect, we should see *much* fewer than 5 % of runs returning a discovery. If the observed frequency of “found” regions is notably above $\alpha^{K}$, that suggests some residual correlation across folds or potential mis-specification of the selection logic. In any case, large deviations from this (very small) nominal rate would indicate that the split criterion or subsequent inference may be more permissive than intended under strictly null conditions.

### 8.4 Evaluating performance

To assess the ability of CVtreeMLE to recover an *oracle region* and accurately estimate the corresponding ARE, we conducted the simulation experiments explained in the previous section. Below, we describe our study design and the metrics used to quantify performance. Additionally, for the discrete case, we also evaluate the performance of CVtreeMLE to estimate the data-adaptive ARE, that is, when the discovered threshold is applied to $P_{0}$ how much our estimated ARE in that region deviates from the true ARE when the threshold is applied to $P_{0}$.

#### 8.4.1 Simulation design

For each sample size n (e.g., 200, 500, 1,000, 2,000, 5,000), we replicated the fitting procedure 300 times to get estimates for independent datasets. Each simulation replicate proceeds as follows:

1. **Generate a Dataset:** Sample n i.i.d. observations from the DGP.
2. **Cross-Fitting:** Perform K-fold cross-validation. In each fold, a training partition estimates the threshold-based region ${\overset{ˆ}{𝒜}}_{n_{-k}}$ (via our decision tree procedure) and nuisance functions ($\hat{Q},\hat{g}$), which are then applied to the validation partition for TMLE.
3. **Pooled TMLE Estimate:** Combine all fold-specific estimates into a pooled TMLE estimator ${\hat{\psi }}_{\text{pooled}}$ for the ARE, restricted to ${\overset{ˆ}{𝒜}}_{n}$ (the final, combined region).
4. **Evaluation Against Oracle:** Compare $\left({\overset{ˆ}{𝒜}}_{n},{\overset{ˆ}{\psi }}_{\text{pooled}}\right)$ to the oracle pair $\left({𝒜}^{*},\psi_{0}\right)$, where $\psi_{0}$ denotes the true ARE.

#### 8.4.2 Metrics

- **Region Identification:** We treat ${\overset{ˆ}{𝒜}}_{n}$ and ${𝒜}^{*}$ as subsets of the exposure space and compute:
(1) $$\text{Sensitivity}=\frac{\text{True}\;\text{Positives}}{\text{True}\;\text{Positives}+\text{False}\;\text{Negatives}},\;\text{Specificity}=\frac{\text{True}\;\text{Negatives}}{\text{True}\;\text{Negatives}+\text{False}\;\text{Positives}},\;\text{Accuracy}=\frac{\text{True}\;\text{Positives}+\text{True}\;\text{Negatives}}{\text{Total}\;\text{Population}}.$$
  Here, “true positives” means observations correctly classified inside ${𝒜}^{*}$, etc.
- **Absolute Bias and Percent Bias (ARE):** For each replicate, let ${\hat{\psi }}_{\text{pooled}}$ be the estimated ARE and $\psi_{0}$ the true ARE. Then:
$$\text{Absolute}\;\text{Bias}=\left|{\hat{\psi }}_{\text{pooled}}-\psi_{0}\right|,\;\text{Percent}\;\text{Bias}=100\times \frac{{\hat{\psi }}_{\text{pooled}}-\psi_{0}}{\psi_{0}}.$$
- **MSE and Coverage:** We let
$$\text{MSE}={\left({\hat{\psi }}_{\text{pooled}}-\psi_{0}\right)}^{2}+\text{Var}({\hat{\psi }}_{\text{pooled}}),$$
  and compute the empirical 95 % coverage by checking the proportion of confidence intervals that contain $\psi_{0}$.

In the following section, we present the simulation results, illustrating how CVtreeMLE performs under a variety of sample sizes and data-generating scenarios.

### 8.5 Default estimators

As discussed, CVtreeMLE needs estimators for Q=E(Y∣A,W) and g=P(A∣W). CVtreeMLE has built in default algorithms to be used in a Super Learner [26] that are fast and flexible. These include random forest, general linear models, elastic net, and xgboost. These are used to create Super Learners for $Q_{n}$ and $g_{n}$. Users can pass in their own libraries for these nuisance and data-adaptive parameters. For these simulations, we use these default estimators in each Super Learner. Our partitioning algorithm to discover the region 𝒜 was set to have a maximum depth of 3 and a minimum number of observations per leaf to 10. In this simulation we are interested in finding the region with maximum outcome. The package has a “min_max” parameter which we set to “max” to find the region with maximum expected mean outcome. We used the greedy causal tree search algorithm to test if the greedy approach successfully identifies the oracle region in various data generating systems.

### 8.6 Results

#### 8.6.1 Discrete exposure simulation: CVtreeMLE recovers the oracle region and estimates its effect

**Figure 1:**
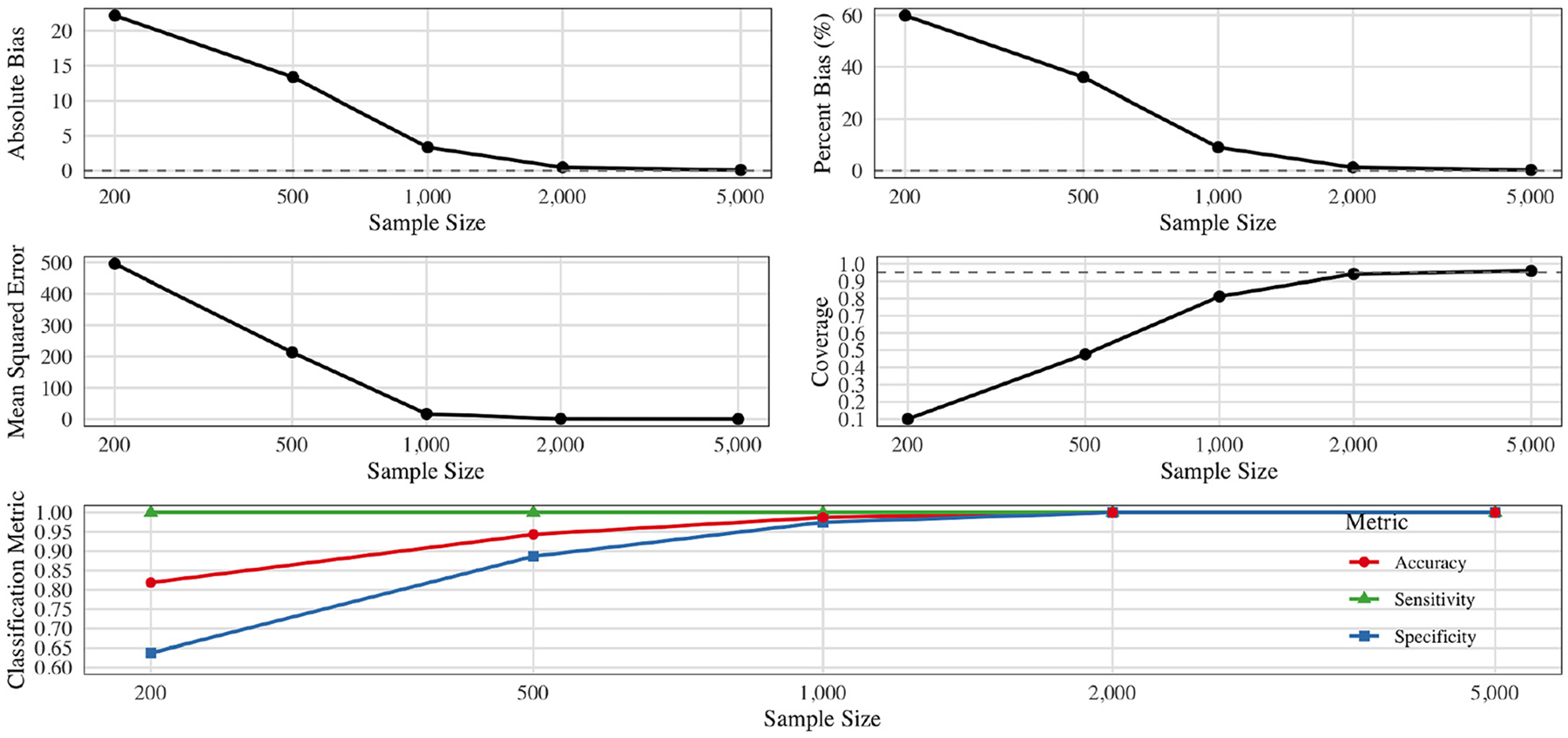
Discrete exposure simulation findings for the oracle parameter.

Figure 1 presents findings for a discrete-exposure scenario in which there is a true “oracle” subregion (involving an interaction of two exposure variables) that maximizes the outcome under positivity constraints. At lower sample sizes (e.g. n=300), the algorithm cannot always select the exact oracle region because it must maintain enough positivity for valid inference. In particular, the tree often identifies a nearby, slightly less extreme region with sufficient probability mass. This manifests as higher bias and reduced coverage for both the region classification (higher false-positives/negatives) and the corresponding attributable regional effect (ARE) estimate.

As n increases, however, the method converges to the ground-truth subregion, as more samples satisfy the positivity requirement while still capturing the true threshold boundary. In turn, both absolute and percent bias for the estimated ARE diminish to near zero, and coverage rises to 95 %. The classification metrics (accuracy, sensitivity, specificity) likewise demonstrate improvement from moderate levels at n=200 to virtually perfect classification by n=2,000. Put differently, once the tree can pinpoint the oracle threshold within a well-supported subregion, the effect estimates become nearly unbiased, achieving nominal coverage and very small mean-squared error. These results highlight how CVtreeMLE, even with simple greedy splits, recovers the true thresholding regime under realistic positivity constraints, provided the sample size is sufficient to ensure all relevant exposures and interactions appear with nontrivial frequency.

#### 8.6.2 Performance for the data-adaptive rule applied to the true population

**Figure 2:**
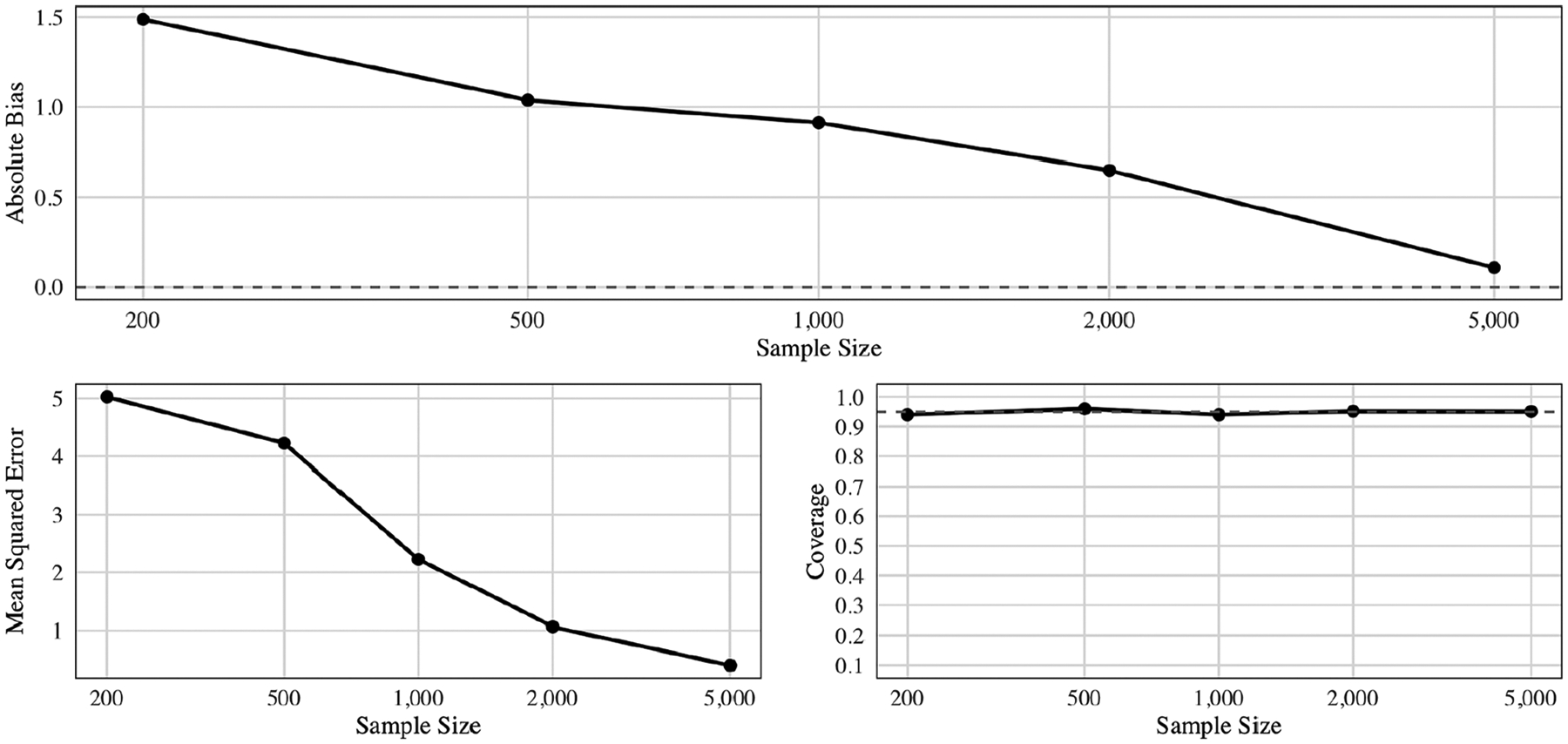
Bias, MSE and coverage for the data-adaptive parameter.

Figure 2 illustrates the absolute bias, mean squared error (MSE), and coverage for the ARE under a *data-adaptive* rule – that is, the region discovered by CVtreeMLE in each sample – applied to the true population $P_{0}$. Unlike the “oracle-based” analysis (Section 8.6.1), where we evaluated the effect of a known, exact threshold rule, here we assess how accurately our *estimated* rule represents the best practical intervention in the limit as n grows.

When n is small (e.g., n=200), the estimated ARE for the selected rule deviates more from the true ARE when the threshold boundary is applied to the population, this is due to finite-sample constraints and random sampling variation, leading to higher bias (≈1.5) and an MSE (≈5). As n increases, however, the discovered data-adaptive region converges to the same policy one would choose with full knowledge of $P_{0}$, thereby aligning the estimated ARE with the true population-level counterfactual effect. Accordingly, bias declines by more than an order of magnitude from n=200 to n=5,000, and the MSE drops from 5.03 to 0.4. Coverage is consistently at or close to 95 %. In short, these results confirm that CVtreeMLE not only estimates the correct *oracle* rule with increasing accuracy, but also yields valid inferences for the target parameter “ARE of the discovered rule” under the true population distribution.

### 8.7 Results for the continuous exposure simulation

**Figure 3:**
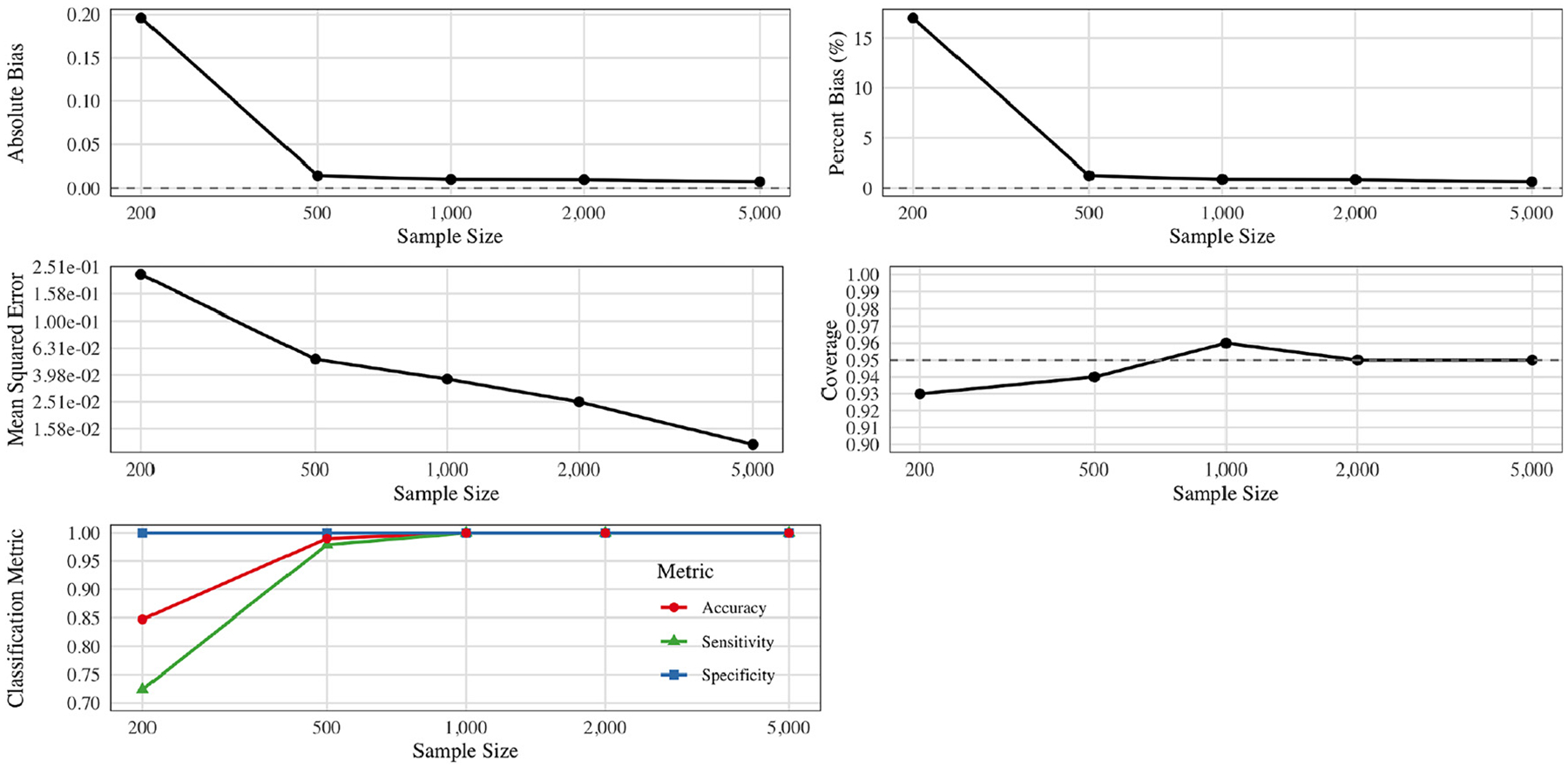
Continuous exposure simulation findings.

Figure 3 shows the estimation performance for the simulation with two correlated, rounded continuous exposures $\left(A_{1},A_{2}\right)$. We considered sample sizes n∈{200,500,1,000,2,000,5,000}, each replicated 300 times. When n is small (e.g., n=200), the procedure does not always discover the true threshold region ($A_{1}>0.5,A_{2}<-0.2$), leading to higher bias and slightly lower coverage. As n grows, however, the estimated region converges to the oracle region, dramatically reducing *both* the absolute and percent bias.

In particular, absolute bias falls from roughly 0.20 at n=200 to near zero by n=5,000. The mean squared error (MSE) decreases quickly (from about 0.219 at n=200 to 0.01 at n=5,000), reflecting both variance reduction and bias elimination. Coverage begins just under the nominal 95 % at n=200 and stabilizes close to or just above 95 % for n≥1,000.

Finally, the classification metrics illustrate how effectively the algorithm identifies whether each observation’s exposures lie inside or outside the true region. Specificity is nearly 100 % at all sample sizes, while sensitivity and overall accuracy rise from about 70–85 % at n=200 to nearly 100 % by n=1,000. Taken together, these findings demonstrate that once the threshold region is accurately recovered, the corresponding effect estimates achieve negligible bias, small MSE, and nominal coverage, underscoring the reliable performance of our data-adaptive approach in continuous-exposure settings.

#### 8.7.1 Null simulation results

At sample sizes n=200 through n=1,000, the algorithm reported *no discovered region* in *all* (100 %) simulation runs. This reflects the conservativeness of our procedure under the strict null, as the cross-validation criterion requires each fold to find a significant subregion for a global region to be declared. At n=2,000, about 96 % of runs did not discover a region, of the 4 % that did *none* excluded zero in the confidence interval. The mean estimated effect in these instances was roughly −0.644, with zero consistently included in all CIs. At n=5,000 a region passed the initial split test in 95 % of replications, yet none of the pooled TMLE estimates were significant (mean ARE = 0.001, 95 % CI [−0.012, 0.014] across 300 runs). Thus all confidence intervals still covered the null, confirming strict control of Type I error.

##### Interpretation.

These findings confirm that CVtreeMLE remains *highly conservative* when there is genuinely no relationship between the exposures and the outcome. Even for moderate sample sizes, it typically finds no region or, if a subregion is discovered, its estimated effect is not significantly different from zero. This conservatism is largely a consequence of our cross-validation rule, which declares a region only if every fold independently flags a significant improvement. Hence, under the null, the global false-positive rate is effectively reduced to the order of $\alpha^{K}$, making spurious discoveries extremely unlikely.

## 9 Applications

### 9.1 NIEHS synthetic mixtures

The NIEHS synthetic mixture dataset (found here on github) is a widely used benchmark for evaluating statistical methods that analyze chemical mixtures. These data can be viewed as originating from a prospective cohort study, where the outcome cannot affect the exposures (as might happen in a cross-sectional design). Correlations between exposures arise from shared sources or modes of exposure. The covariate W (a binary variable here) is intended to act as a potential confounder rather than a collider.

#### 9.1.1 Data structure

There are seven continuous exposures, denoted $A_{1},\ldots ,A_{7}$, each of which can influence an endocrine-disruption outcome Y through either synergistic or antagonistic pathways. Specifically:

- $A_{1},A_{2},A_{7}$ *increase* Y (positive coefficients).
- $A_{4},A_{5}$ *decrease* Y (negative coefficients).
- $A_{3},A_{6}$ have no net effect on Y, but remain correlated with other exposures, making them challenging to discard in a purely statistical screen.

In this synthetic setup, the seven exposures are further grouped by chemical or functional similarities. For instance, ($A_{1},A_{2},A_{3}$) form a cluster, while ($A_{5},A_{6}$) form another. Each cluster shows high internal correlation, reflecting plausible biological scenarios (e.g., multiple polychlorinated biphenyl congeners that are chemically related yet differ in toxicity).

#### 9.1.2 Toxicological background

These data exhibit a variety of dose-response patterns and interactions:

- $A_{1}$ and $A_{2}$ combine additively to raise Y.
- $A_{4}$ competes with $A_{1}$ or $A_{2}$ (“competitive antagonism”), partially offsetting their positive effect.
- $A_{1}-A_{7}$ and $A_{2}-A_{7}$ can be synergistic (supra-additive), intensifying the increase in Y.
- $A_{4}$ and $A_{5}$ each lower Y in an additive fashion, while $A_{5}-A_{7}$ can exhibit “unusual antagonism.”

Such structural assumptions echo real-world toxicology, where chemically related exposures may be correlated and yield complex interactions.

#### 9.1.3 Analysis goal

We consider 500 observations and nine variables (the seven exposures plus W and Y). Our aim is to apply CVtreeMLE to identify *threshold-based subregions* of $\left(A_{1},\ldots ,A_{7}\right)$ that *minimize* the outcome Y. Constraining exposures to these thresholds should, in principle, yield the largest reduction in Y. We used 10-fold cross-validation and default Super Learner libraries for the nuisance parameters, running the algorithm in parallel to assess computational feasibility.

#### 9.1.4 Fold-specific results

Table 1 shows, for each fold, the discovered subregion associated with the strongest negative attributable regional effect (ARE) on Y. Most folds (70 %) favored a policy that forces $X_{5}$ above approximately 1.9–2.0, reflecting $A_{5}$’s substantial negative influence on Y. In contrast, two folds (20 %) constrained $X_{7}$ below a threshold (0.3–0.4), consistent with $A_{7}$ being a driver of higher Y. Finally, one fold indicated a joint rule $X_{7}\leq 0.4$ and $X_{1}\leq 0.6$, suggesting an antagonistic interplay among $A_{1}$ and $A_{7}$.

#### 9.1.5 Pooled TMLE results

We then aggregated these fold-specific findings into a pooled TMLE analysis, shown in Table 2. Three subregions consistently emerged:

1. $X_{5}>1.9$ (or 2.0), with an average effect of about −4.6;
2. $X_{7}\leq 0.3$, around −4.5;
3. A joint rule $X_{7}\leq 0.4$ and $X_{1}\leq 0.6$, yielding about −4.1.

All are statistically significant, and the slight numerical differences suggest these thresholds have near-equivalent potential for reducing Y.

#### 9.1.6 Interpretation

Overall, the data-adaptive procedure uncovered *multiple* threshold policies that each lowers Y by roughly 4–5 units. Restricting $X_{7}$ to low levels is intuitive since $A_{7}$ has a positive effect on Y. Conversely, requiring $X_{5}$ to be sufficiently high exploits $A_{5}$’s negative relationship with Y. The joint rule involving $X_{1}$ and $X_{7}$ aligns with their established synergistic relationship built into the data. In practice, regulators or policy analysts might choose among these subregions based on additional criteria – such as feasibility, cost, or tolerable exposure levels – given that all produce similar reductions in the outcome.

Thus, the NIEHS data example illustrates how CVtreeMLE can detect multiple plausible subregions in a complex mixture setting and highlights the interpretability of threshold-based results when exposures interact in realistic ways.

#### 9.1.7 Comparison to existing methods

Quantile g-computation [8], prevalent in environmental epidemiology for mixture analysis, estimates the effects of uniformly increasing exposures by one quantile, based on linear model assumptions. This method quantizes mixture components, summing the linear model’s coefficients to form a summary measure (Ψ) for joint impact assessment. However, it inherently assumes additive, monotonic exposure-response relationships, overlooking complex, potentially nonlinear interactions typical in mixtures, like in endocrine disrupting compounds. Consequently, this method might not accurately capture the nuanced dynamics of mixed exposures, especially when interactions vary with other variable levels.

We run quantile g-computation on the NIEHS data using 4 quantiles with no interactions to investigate results using this model. The size of the scaled effect (positive direction, sum of positive coefficients) was 6.28 and included $A_{1},A_{2},A_{3},A_{7}$ and the scaled effect size (negative direction, sum of negative coefficients) was −3.68 and included $A_{4},A_{5},A_{6}$. Compared to NIEHS ground truth, $A_{3},A_{6}$ are incorrectly included in these estimates. However, the positive and negative associations for the other variables are correct. Next, because we expect interactions to exist in the mixture data, we would like to assess for them but the question is which interaction terms to include? Our best guess is to include interaction terms for all exposures. We do this and show results in Table 3.

In Table 3 ${𝚿}_{1}$ is the summary measure for the main effects and ${𝚿}_{2}$ for interactions. As can be seen, when including all interactions, neither of the estimates are significant. Of course, this is to be expected given the number of parameters in the model and the sample size n=500. However, moving forward with interaction assessment is difficult; if we were to assess for all 2-way interaction of 7 exposures, the number of sets is 21 and with 3-way interactions is 35. We would have to run these many models and then correct for multiple testing. Hopefully, this example shows why mixtures are inherently a data-adaptive problem and why popular methods such as this, although succinct and interpretable, fall short even in a simple synthetic data set.

### 9.2 NHANES data

We illustrate our method on a publicly available subset of the National Health and Nutrition Examination Survey (NHANES) from 1999 to 2002, focusing on urinary metal exposures and leukocyte telomere length (LTL). Telomere length is often investigated as a biomarker of cellular aging, and there is scientific interest in how various toxic metal exposures may shorten or lengthen telomeres. For concreteness, we include this data in CVtreeMLE’s open-source repository, which comprises 2,510 participants with measured LTL, demographic variables, and nine urinary metals: barium, cadmium, cobalt, cesium, molybdenum, lead, antimony, thallium, and tungsten.

We adjust for potential confounders, including age, gender, race, and select lifestyle variables. Our primary goal is to discover a region that *minimizes* LTL (i.e., identifies the “worst” combination of exposures).

#### 9.2.1 Fitting strategy

We applied CVtreeMLE with 10-fold cross-validation. In each fold, the algorithm searched the 9-dimensional exposure space for a subregion that yielded the lowest counterfactual LTL when forcing all individuals into that region, subject to positivity constraints, an α significance level of 0.05 and minimum node size of 25. A Super Learner ensemble (including GLMs and random forests) was used to estimate the nuisance functions $\hat{Q}$ and $\hat{g}$. Afterward, we pooled the fold-specific targeted estimates to obtain an overall Attributable Regional Effect (ARE).

#### 9.2.2 Fold-specific results

Table 4 shows the estimates of the ARE and the discovered region in each fold. Notably, CVtreeMLE consistently selected a threshold on *molybdenum* (ranging between roughly 96.7 and 112.4) as the region that minimizes LTL. No fold identified a meaningful threshold interaction involving other metals. Each fold-specific point estimate was near zero, with confidence intervals consistently covering zero, implying no significant effect, although the fact molybdenum being selected as the minimizer for all folds suggest there is some positive association signal for this exposure.

#### 9.2.3 Pooled ARE estimate

We then pooled all fold-specific estimates via cross-validated TMLE. As summarized in Table 5, restricting molybdenum to a threshold near 103.8 (the average discovered cut point) yields a pooled ARE estimate of −0.002, suggesting a minimal decrease in average LTL. The 95 % confidence interval (−0.006, 0.003) covers zero, indicating no statistically significant difference from the observed mean telomere length.

#### 9.2.4 Interpretation

Our analysis did not reveal any strong evidence that restricting multiple metal exposures simultaneously would meaningfully decrease LTL. Instead, across folds, a single threshold on molybdenum emerged as the candidate “lowest LTL” region. However, the effect size is small, and the confidence intervals comfortably include zero.

These findings may diverge from some prior studies suggesting that molybdenum could affect telomere attrition. Differences might arise from population characteristics, the cross-sectional nature of this study, sample sizes, or unmeasured confounding. From a mixtures perspective, it is noteworthy that no other metal (e.g., cadmium, lead, barium) had a consistently discovered threshold, indicating that in this data set, limiting molybdenum alone provided the best – though statistically insignificant – candidate for reducing telomere length.

Overall, CVtreeMLE systematically searched a 9-dimensional exposure space using data-adaptive thresholds, returning an easily interpretable subregion and valid inference. Despite the absence of a strong signal here, this example highlights how the procedure can rigorously explore potential mixture effects in real-world environmental data.

## 10 Discussion

This paper introduces a data-adaptive procedure for identifying threshold-based subregions in high-dimensional exposure spaces and estimating the effects of hypothetical policies that restrict populations to those subregions. By defining the attributable regional effect (ARE) as a policy-oriented causal parameter and combining recursive partitioning with cross-validated targeted maximum likelihood estimation (CV-TMLE), we can discover interpretable “cut points” in multivariate exposures while maintaining statistical rigor.

### 10.1 Key contributions

Our method relaxes many of the strong assumptions commonly employed in mixture analysis. Unlike regression-based approaches such as weighted quantile sum (WQS) regression [8] or Bayesian kernel machine regression (BKMR) [10], our approach directly targets the effect of restricting exposures to data-determined subregions. In doing so, we leverage data-adaptive threshold discovery – ensuring that critical exposure boundaries reflect observed empirical patterns rather than *a priori* specifications. Moreover, our significance filtering at each split (Section 5.1) naturally guards against overfitting, while cross-validation (Section 6) yields valid inference despite reusing data for threshold selection and effect estimation.

### 10.2 Practical relevance for policy

From a policy standpoint, the discovered region can be interpreted as a candidate set of allowable exposures (or a “safety threshold”) that the population could be *forced* or encouraged to adhere to. By preserving relative selection densities within that region, we minimize modeling assumptions about how individuals might shift their exposure choices. Although such self-selection may not fully mirror how real regulations operate (e.g., individuals clustering below a boundary), it offers a transparent starting point for quantifying public-health gains. As our simulations and examples demonstrate, even simple bounding thresholds in multiple exposures can uncover meaningful interactions (*e.g*., synergy between $X_{1}$ and $X_{7}$ in the NIEHS data) or harmful univariate thresholds (molybdenum in the NHANES data).

### 10.3 Limitations and future directions

While our approach shows promise, several challenges remain:

1. **Fold-to-Fold Variability in Thresholds:** If different cross-validation folds discover substantially different partitions, this suggests weaker underlying signals or high sensitivity to sampling variation. The responsibility lies with the practitioner to report all fold findings, thereby being transparent with consistency rather than picking folds to report that match preconceived notions.
2. **Positivity Constraints:** By design, we discard splits that lead to near-zero probability for certain covariate strata. While this mitigates overfitting, it also excludes extremely sparse exposures that might be biologically relevant. More adaptive positivity thresholds (varying by sample size or exposure dimension) and advanced smoothing strategies may offer improvements.
3. **High-Dimensional Scaling:** In settings with tens or hundreds of exposures, enumerating all candidate cut points is computationally onerous. Potential solutions include domain-based grouping (binning or combining chemically similar exposures), dimension reduction, or hierarchical Bayesian priors tailored to known toxicologic relationships. Future work may explore using more advanced search algorithm like neural network guided monte-carlo tree search algorithms [27–29].
4. **Alternative Policy Interpretations:** We rely on the assumption of *relative self-selection* to define post-intervention exposures, yet real-world policies might concentrate populations near a boundary or reconfigure entire distributions. Incorporating domain knowledge of how exposures shift (e.g., partial or gradient-based compliance) stands as an important frontier for more realistic policy modeling.

### 10.4 Conclusions

Overall, this work adds to a growing literature on data-adaptive causal inference in environmental epidemiology. Our CVtreeMLE framework blends flexible machine learning and valid semiparametric inference with an explicitly policy-focused parameter: the attributable regional effect. Through theoretical analysis, simulations, and applications to NIEHS synthetic mixtures and NHANES metal data, we demonstrate that our method can discover threshold-based multi-exposure subsets that meaningfully alter population-level outcomes. Methodological enhancements, including stability diagnostics and algorithms that handle extremely high-dimensional exposures, promise to further strengthen the real-world applicability of threshold-based exposure interventions. We anticipate that such developments, combined with domain-specific insights, will make data-adaptive threshold inference ever more useful for guiding public health and regulatory decisions.

## Figures and Tables

**Table 1:** Fold-specific subregions minimizing Y in the NIEHS synthetic data.

Fold	Region	ARE	SE	95 % CI	p-value	Variables
1	$X_{5}>2$	−3.824	0.574	(−4.949, −2.699)	< 0.0001	X5
2	$X_{7}\leq 0.3$	−3.859	0.586	(−5.007, −2.710)	< 0.0001	X7
3	$X_{5}>1.9$	−4.268	2.091	(−8.366, −0.170)	0.0412	X5
4	$X_{7}\leq 0.3$	−5.101	0.641	(−6.356, −3.845)	< 0.0001	X7
5	$X_{5}>2$	−4.628	2.214	(−8.967, −0.289)	0.0366	X5
6	$X_{5}>2$	−4.663	2.185	(−8.945, −0.381)	0.0328	X5
7	$X_{7}\leq 0.4\&X_{1}\leq 0.6$	−4.122	0.608	(−5.314, −2.930)	< 0.0001	X7-X1
8	$X_{5}>2$	−4.727	1.612	(−7.885, −1.568)	0.00336	X5
9	$X_{5}>1.9$	−5.157	0.486	(−6.110, −4.204)	< 0.0001	X5
10	$X_{5}>1.9$	−4.646	1 .763	(−8.101, −1.192)	0.00839	X5

ARE denotes the estimated change in Y from forcing everyone into the specified region, with 95 % confidence intervals and p-values based on fold-specific influence-function estimates.

**Table 2:** Pooled TMLE estimates for discovered subregions.

Solution	Mixture ATE	SE	95 % CI	p-value	Region	Prop. folds
(1) X5	−4.559	0.641	(−5.815, −3.303)	< 0.0001	$X_{5}>1.957(1.9,2.0)$	0.70
(2) X7	−4.480	0.442	(−5.347, −3.613)	< 0.0001	$X_{7}\leq 0.3$	0.20
(3) X7-X1	−4.122	0.608	(−5.314, −2.930)	< 0.0001	$X_{7}\leq 0.4,X_{1}\leq 0.6$	0.10

“Proportion Folds” denotes how often each solution was selected among the 10 folds. “Mixture ATE” is the estimated change in Y (ARE).

**Table 3:** Quantile G-computation interaction results from NIEHS synthetic data.

	Estimate	Std. error	Lower CI	Upper CI	Pr(>|t|)
(Intercept)	21.29	1.58	18.19	24.39	0.00
psi1	0.02	1.62	−3.16	3.20	0.99
psi2	0.59	0.67	−0.71	1 .90	0.37

**Table 4:** Fold-specific ARE estimates for urinary metals versus telomere length.

Fold	ARE	SE	Lower CI	Upper CI	p-value	Region
1	0.000	0.003	−0.006	0.006	1 .000	molybdenum ≤ 102.9
2	−0.001	0.003	−0.006	0.004	0.670	molybdenum ≤ 103.6
3	−0.001	0.003	−0.006	0.005	0.845	molybdenum ≤ 104.5
4	−0.001	0.002	−0.004	0.002	0.597	molybdenum ≤ 112.4
5	−0.002	0.003	−0.007	0.003	0.409	molybdenum ≤ 107.3
6	−0.005	0.003	−0.011	0.001	0.141	molybdenum ≤ 102.9
7	0.000	0.008	−0.017	0.016	0.981	molybdenum ≤ 104.9
8	−0.003	0.004	−0.010	0.005	0.440	molybdenum ≤ 96.7
9	−0.002	0.003	−0.008	0.004	0.471	molybdenum ≤ 100.2
10	−0.001	0.003	−0.007	0.004	0.657	molybdenum ≤ 102.5

The region in each fold restricts molybdenum to the specified threshold. All estimates are near zero, with no significance.

**Table 5:** Pooled TMLE ARE for the discovered region (molybdenum ≤ 103.8 (96.7, 112.4)).

ARE	Std. error	Lower CI	Upper CI	p-value	Region
−0.002	0.002	−0.006	0.003	0.468	molybdenum ≤103.8 (96.7, 112.4)

## References

[1] Landrigan PJ, Goldman LR. Children’s health and the environment: public health implications of exposures to neurotoxic chemicals in early childhood. Pediatrics 2006;118:259–67.

[2] Vrijheid M, Slama R, van den Brandt PA. Mixtures of environmental exposures and health: challenges and opportunities for environmental epidemiology. Int J Environ Res Publ Health 2009;6:205–23.

[3] Kortenkamp A Low-dose mixture effects of endocrine disrupters: implications for risk assessment and human health. Environ Health Perspect 2014;122:A10–15.24380927

[4] Braun JM, Gennings C. The emerging science of environmental mixtures and the future of risk assessment. Environ Health Perspect 2018; 126:128–34.

[5] National Research Council. Cumulative environmental risks: building an integrated framework to better inform decision-making. Washington, DC: National Academies Press; 2018.

[6] NRC. Phthalates and cumulative risk assessment: the tasks ahead. Washington, DC: National Academies Press; 2008. 25009926

[7] Carlin DJ, Rider CV, Woychik R, Birnbaum LS. Unraveling the health effects of environmental mixtures: an niehs priority. Environ Health Perspect 2013;121:A6–8. 23409283 10.1289/ehp.1206182 PMC3553446

[8] Keil AP, Buckley JP, O’Brien KM, Ferguson KK, Zhao S, White AJ. A quantile-based g-computation approach to addressing the effects of exposure mixtures. arXiv 2019;128:1–10. 10.1289/EHP5838 PMC7228100 32255670

[9] De Vocht F, Cherry N, Wakefield J. A Bayesian mixture modeling approach for assessing the effects of correlated exposures in case-control studies. J Expo Sci Environ Epidemiol 2012;22:352–60. 22588215 10.1038/jes.2012.22

[10] Bobb JF, Valeri L, Henn BC, Christiani DC, Wright RO, Mazumdar M, Bayesian kernel machine regression for estimating the health effects of multi-pollutant mixtures. Biostatistics 2014;16:493–508. 25532525 10.1093/biostatistics/kxu058 PMC5963470

[11] van der Laan MJ, Rose S. Targeted learning: causal inference for observational and experimental data. Springer Series in Statistics. Springer New York; 2011. Available from: https://books.google.com/books?id=RGnSX5aCAgQC.

[12] van der Laan MJ, Rose S. Targeted learning in data science: causal inference for complex longitudinal studies. Springer Series in Statistics. Springer International Publishing; 2018. Available from: https://books.google.com/books?id=vKFTDwAAQBAJ.

[13] Zheng W, van der Laan MJ. Asymptotic theory for cross-validated targeted maximum likelihood estimation. U.C. Berkeley Division of Biostatistics Working Paper Series, (273); 2010. Available from: http://biostats.bepress.com/ucbbiostat/paper273/.

[14] Hubbard AE, Kherad-Pajouh S, Van Der Laan MJ. Statistical inference for data adaptive target parameters. Int J Biostat 2016;12:3–19. 27227715 10.1515/ijb-2015-0013

[15] McCoy D, Hubbard A, Van der Laan M. Cvtreemle: efficient estimation of mixed exposures using data adaptive decision trees and cross-validated targeted maximum likelihood estimation in r. J Open Source Softw 2023;8:4181. 37398941 10.21105/joss.04181 PMC10312067

[16] Li H, Rosete S, Coyle J, Phillips RV, Hejazi NS, Malenica I, Evaluating the robustness of targeted maximum likelihood estimators via realistic simulations in nutrition intervention trials. Stat Med 2022;41:2132–65. 35172378 10.1002/sim.9348 PMC10362909

[17] Breiman L, Friedman J, Olshen R, Stone C. Classification and regression trees. Belmont, CA: Wadsworth International Group; 1984.

[18] Hothorn T, Hornik K, Zeileis A. Unbiased recursive partitioning: a conditional inference framework. J Comput Graph Stat 2006;15:651–74.

[19] Athey S, Imbens GW. Recursive partitioning for heterogeneous causal effects. Proc Natl Acad Sci 2016;113:7353–60. 27382149 10.1073/pnas.1510489113 PMC4941430

[20] Wager S, Athey S. Estimation and inference of heterogeneous treatment effects using random forests. JAm Stat Assoc 2018;113:1228–42. First posted as arXiv:1510.04342 [stat.ME] in 2015.

[21] Hubbard AE, Kennedy CJ, van der Laan MJ. Data-adaptive target parameters. In: van der Laan MJ, Rose S, editors. Targeted learning in data science, Springer Series in Statistics. Cham, Switzerland: Springer; 2018:125–42 pp.

[22] Bertsimas D, Dunn J. Optimal classification trees. Mach Learn 2017;106:1039–82. First posted as arXiv:1702.01761 [cs.LG].

[23] Hu B, Rudin C, Seltzer M. Optimal decision trees. Oper Res 2020;68:1517–37. First posted as arXiv:1810.05041 [cs.LG].

[24] Lage IJ, Sudjianto A, Zeng X, Hooker G. A general framework for globally and locally optimal decision trees. In: Proceedings of the 39th international conference on machine learning (ICML); 2022:11942–59 pp. First posted as arXiv:2204.10053 [cs.LG].

[25] van der Vaart AW. Asymptotic statistics. Cambridge, UK: Cambridge University Press; 1998.

[26] van der Laan MJ, Polley EC, Hubbard AE. Super learner. Stat Appl Genet Mol Biol 2007;6:1–23. 10.2202/1544-6115.1309 17910531

[27] Silver D, Huang A, Maddison CJ, Guez A, Sifre L, Van Den Driessche G, Mastering the game of go with deep neural networks and tree search. Nature 2016;529:484–9. 26819042 10.1038/nature16961

[28] Silver D, Schrittwieser J, Simonyan K, Antonoglou I, Huang A, Guez A, Mastering chess and shogi by self-play with a general reinforcement learning algorithm. arXiv preprint arXiv:1712.01815 2017.

[29] Silver D, Schrittwieser J, Simonyan K, Antonoglou I, Huang A, Guez A, Mastering the game of go without human knowledge. Nature 2017;550:354–9. 29052630 10.1038/nature24270

